# Sex Differences in Animal Models of Traumatic Brain Injury

**DOI:** 10.1177/1179069519844020

**Published:** 2019-05-13

**Authors:** Todd G Rubin, Michael L Lipton

**Affiliations:** 1 The Dominick P. Purpura Department of Neuroscience, Albert Einstein College of Medicine, Rose F. Kennedy Center, Bronx, NY, USA; 2 Gruss Magnetic Resonance Research Center, Albert Einstein College of Medicine, Bronx, NY, USA; 3 Department of Radiology, Albert Einstein College of Medicine, Montefiore Medical Center, Bronx NY, USA; 4 Department of Psychiatry and Behavioral Sciences, Albert Einstein College of Medicine, Bronx, NY, USA

**Keywords:** Cognition, neuropathology, neurotrauma, neuroimaging, murine

## Abstract

Traumatic brain injury (TBI) is highly prevalent and there is currently no adequate treatment. Understanding the underlying mechanisms governing TBI and recovery remains an elusive goal. The heterogeneous nature of injury and individual’s response to injury have made understanding risk and susceptibility to TBI of great importance. Epidemiologic studies have provided evidence of sex-dependent differences following TBI. However, preclinical models of injury have largely focused on adult male animals. Here, we review 50 studies that have investigated TBI in both sexes using animal models. Results from these studies are highly variable and model dependent, but largely show females to have a protective advantage in behavioral outcomes and pathology following TBI. Further research of both sexes using newer models that better recapitulate mild and repetitive TBI is needed to characterize the nature of sex-dependent injury and recovery, and ultimately identifies targets for enhanced recovery.

## Introduction

Traumatic brain injury (TBI) is a major public health burden. The number of TBIs per year is not known due to the many that go undiagnosed, but it has increased steadily over the past decade and is estimated that anywhere from 1.4 to 42 million occur every year.^1–4^

Most head trauma, up to 80% of all cases, fall into the mild subgroup of traumatic brain injury (mTBI), also known as concussion.^5,6^ Concussion occurs from a direct blow to or rapid acceleration-deceleration of the head, with or without loss of consciousness, that causes rapid onset of altered neurological function. Typical symptoms include headache, nausea, sensitivity to light, and impaired concentration and memory.^7^ Although concussion produces symptoms that are understood to arise from brain pathology, clinical imaging abnormalities such as hemorrhage or skull fracture are not typically present.^8,9^ The diverse mechanisms by which concussion occurs and its varied clinical symptoms implicate heterogeneity of the underlying neuropathology.^10^ Although most patients recover from concussion in a matter of hours to weeks, some remain symptomatic for months or even years.^11–13^

Understanding why some patients recover relatively quickly whereas others remain symptomatic is of the utmost importance. Preclinical studies over the past 30 years have yielded valuable information about various aspects of injury and recovery, such as the elaboration of a prolonged innate neuroinflammatory process and acute changes in phosphorylated tau deposition following concussion.^14–16^ Currently, the only clinical treatment for concussion is rest and supportive care. Because strict rest may actually confer worse and prolonged symptoms, active recovery methods have started to be implemented.^17–19^ In the search for direct biological interventions to treat concussion, animal models are critical for defining viable therapeutic targets for ameliorating symptoms and enhancing recovery. Given the heterogeneity with which concussion manifests in humans, it is important for animal models to address potential sources of inter-individual variation.

One area that has received increased attention as a possible modifier of outcome after concussion is biological sex. Multiple studies have shown that females are at greater risk than men for poor outcomes following concussion.^20–23^ Despite the fact that men are at greater risk for concussion due to greater participation in high-risk activities, women tend to report more symptoms and more persistent sequelae following concussion.^22–25^ These findings have been debated due to the subjective self-reporting involved to collect these measures, with some attributing the differences to societal pressure causing men to underreport symptoms.^26^ Various mechanisms have been postulated as to why men and women have different outcomes following concussion and subconcussive injury, such as force of injury, number of injuries, skull and brain shape, neck strength, and hormonal influences.^27,28^ Further investigation, including in animal models, will be necessary to isolate individual aspects of injury and recovery to develop new modes and methods for treatment.

With a growing body of clinical evidence that sex may be an important factor conferring risk from TBI, defining the underlying mechanisms using animal models remains an important goal. This review seeks to summarize the sex-dependent findings from animal models of TBI to better understand what role sex differences play, and where opportunity may lie for future research to identify actionable targets for intervention.

## Methods

This review used a structured search of PubMed to include all relevant articles through 2018. Search terms included “Sex Characteristics” or “Sex Factors”; “Disease Models. Animal”; and “Brain Injuries,” “Concussion,” “TBI,” and “mTBI.” Searches were limited to articles in English. Papers were screened to retain original research articles reporting sex effects related to TBI in animal models. Studies of stroke or other non-TBI injury models such as direct lesion, as well as reviews, editorials, letters and case reports, were excluded. References of included papers were reviewed to identify additional papers. A total of 50 articles were identified and are reviewed here (Table 1).

**Table 1.** All papers examining sex differences after TBI.

Year	AUTHORS (First, Last)	Title	Animal	Both Sexes	Model	Reference
1993	(Claire Emerson, Robert Vink)	Estrogen improves biochemical and neurologic outcome following traumatic brain injury in male rats, but not in females	Adult SD Rat	Yes	FPI	55
1993	(Robin Roof, Donald Stein)	Gender influences outcome of brain injury: progesterone plays a protective role	Adult SD Rat	Yes	Trad CCI	59
1996	(Robin Roof, Donald Stein)	Progesterone rapidly decreases brain edema: treatment delayed up to 24 hours is still effective	Adult SD Rat	Yes	Trad CCI	60
2000	(Robin Roof, Donald Stein)	Estrogen-related gender difference in survival rate and cortical blood flow after impact-acceleration head injury in rats	Adult SD Rat	Yes	Modified weight drop	53
2001	(Takuji Igarashi, Linda Noble)	Regional vulnerability after traumatic brain injury: gender differences in mice that overexpress human copper, zinc superoxide dismutase	CD-1 Mice	Yes	Trad CCI	46
2002	(Amy Wagner, C. Edward Dixon)	Intervention with environmental enrichment after experimental brain trauma enhances cognitive recovery in male but not female rats	Adult SD Rat	Yes	Trad CCI	40
2003	(Nancy Kupina, Edward Hall)	Cytoskeletal protein degradation and neurodegeneration evolves differently in males and females following experimental head injury	CF-1 Mice	Yes	Weight drop	54
2003	(Christine O’Connor, Robert Vink)	Interaction between anesthesia, gender, and functional outcome task following diffuse traumatic brain injury in rats	Adult SD Rat	Yes	Weight drop	29
2003	(Takamoto Suzuki, W. Dalton Dietrich)	The importance of gender on the beneficial effects of posttraumatic hypothermia	Adult SD Rat	Yes	FPI	47
2005	(X Chen, AK Wagner)	Gender and environmental effects on regional brain-derived neurotrophic factor expression after experimental traumatic brain injury	Adult SD Rat	Yes	Trad CCI	81
2005	(Edward Hall, Kirstina Pavel)	Lack of a gender difference in post-traumatic neurodegeneration in the mouse controlled cortical impact injury model	CF-1 Mice	Yes*	Focal CCI	56
2005	(Nigel Jones, Sean Murphy)	The neuroprotective effect of progesterone after traumatic brain injury in male mice is independent of both the inflammatory response and growth factor expression	Adult C57	Yes	Aseptic cryogenic cerebral injury	48
2005	(Christine O’Connor, Robert Vink)	Both estrogen and progesterone attenuate edema formation following diffuse traumatic brain injury in rats	Adult SD Rat	Yes**	Weight drop	62
2005	(Changsheng Qu, Michael Chopp)	Effect of atorvastatin on spatial memory, neuronal survival, and vascular density in female rats after traumatic brain injury	Adult SD Rat	Only females	Trad CCI	49
2005	(Amy Wagner, C. Edward Dixon)	Gender and environmental enrichment impact dopamine transporter expression after experimental traumatic brain injury	SD rat	Yes	Trad CCI	82
2006	(Christine O’Connor, Robert Vink)	The temporal profile of edema formation differs between male and female rats following diffuse traumatic brain injury	Adult SD Rat	Yes	Weight drop	61
2007	(Annadora J Bruce-Keller, Stephen Scheff)	Gender and estrogen manipulation do not affect traumatic brain injury in mice	Adult C57	Yes	Trad CCI	78
2007	(William Armstead, Monica Vavilala)	Adrenomedullin reduces gender-dependent loss of hypotensive cerebrovasodilation after newborn brain injury through activation of ATP-dependent K channels	Piglet	Yes	FPI	67
2007	(D Meffre, R Guennoun)	Steroid profiling in brain and plasma of male and pseudopregnant female rats after traumatic brain injury: analysis by gas chromatography/mass spectrometry	Adult SD Rat	Yes***	Trad CCI	84
2007	(Christine O’Connor, Robert Vink)	Effects of progesterone on neurologic and morphologic outcome following diffuse traumatic brain injury in rats	Adult SD Rat	Yes**	Weight drop	39
2007	(Amy Wagner, C. Edward Dixon)	Gender associations with chronic methylphenidate treatment and behavioral performance following experimental traumatic brain injury	Adult SD Rat	Yes	Trad CCI	33
2007	(Ye Xiong, Michael Chopp)	Role of gender in outcome after traumatic brain injury and therapeutic effect of erythropoietin in mice	Adult C57	Yes	Trad CCI	32
2009	(Helen Bramlett, Robert Keane)	Sex differences in XIAP cleavage after traumatic brain injury in the rat	Adult SD Rat	Yes	FPI	58
2009	(Symeon Missios, Ann-Christine Duhaime)	Scaled cortical impact in immature swine: effect of age and gender on lesion volume	Pigs, 3 ages	Yes	Trad CCI	50
2010	(William Armstead, Monica Vavilala)	Adrenomedullin prevents sex-dependent impairment of autoregulation during hypotension after piglet brain injury through inhibition of ERK MAPK upregulation	Piglet	Yes	FPI	68
2010	(William Armstead, Monica Vavilala)	Impaired cerebral blood flow autoregulation during posttraumatic arterial hypotension after fluid percussion brain injury is prevented by phenylephrine in female but exacerbated in male piglets by extracellular signal-related kinase mitogen-activated protein kinase upregulation	Piglet	Yes	FPI	73
2010	(William Armstead, Monica Vavilala)	SNP improves cerebral hemodynamics during normotension but fails to prevent sex dependent impaired cerebral autoregulation during hypotension after brain injury	Piglet	Yes	FPI	69
2011	(William Armstead, Monica Vavilala)	Phenylephrine infusion prevents impairment of ATP- and calcium-sensitive potassium channel-mediated cerebrovasodilation after brain injury in female, but aggravates impairment in male, piglets through modulation of ERK MAPK upregulation	Piglet	Yes	FPI	70
2011	(Kristin Russell, Beth Levant)	Sensorimotor behavioral tests for use in a juvenile rat model of traumatic brain injury: Assessment of sex differences	Immature LE Rat	Yes	Trad CCI	34
2012	(William Armstead, Monica Vavilala)	TBI sex dependently upregulates ET-1 to impair autoregulation, which is aggravated by phenylephrine in males but is abrogated in females	Piglet	Yes	FPI	71
2013	(William Armstead, Monica Vavilala)	Dopamine prevents impairment of autoregulation after traumatic brain injury in the newborn pig through inhibition of Up-regulation of endothelin-1 and extracellular signal-regulated kinase mitogen-activated protein kinase	Piglet	Yes	FPI	74
2015	(Rachel Lazarus, Gregory Mueller)	Protein carbonylation after traumatic brain injury: cell specificity, regional susceptibility, and gender differences	Adult SD Rat	Yes	Trad CCI	79
2014	(Rebekah Mannix, Shenandoah Robinson)	Sex differences in the effect of progesterone after controlled cortical impact in adolescent mice: a preliminary study	Adolescent C57	Yes	Trad CCI	38
2014	(Richelle Mychasiuk, Michael Esser)	Mean girls: sex differences in the effects of mild traumatic brain injury on the social dynamics of juvenile rat play behaviour	Juvenile SD Rat	Yes	Modified weight drop	35
2015	(Mattias Gunther, Marten Risling)	COX-2 regulation and TUNEL-positive cell death differ between genders in the secondary inflammatory response following experimental penetrating focal brain injury in rats	Adult SD Rat	Yes	Trad CCI^#^	57
2015	(Richelle Mychasiuk, Michael Esser)	A mild traumatic brain injury (mTBI) induces secondary attention-deficit hyperactivity disorder-like symptomology in young rats	Juvenile SD Rat	Yes	Modified weight drop	45
2015	(Courtney Robertson, Manda Saraswati)	Progesterone protects mitochondrial function in a rat model of pediatric traumatic brain injury	Immature SD Rat	Yes	Trad CCI	51
2016	(Rastafa Geddes, Iqbal Sayeed)	Progesterone treatment shows benefit in female rats in a pediatric model of controlled cortical impact injury	P28 SD Rat	Females only^##^	Trad CCI	30
2016	(William Armstead, Monica Vavilala)	Preferential protection of cerebral autoregulation and reduction of hippocampal necrosis with norepinephrine after traumatic brain injury in female piglets	Piglet	Yes	FPI	75
2016	(Richelle Mychasiuk, Michael Esser)	The direction of the acceleration and rotational forces associated with mild traumatic brain injury in rodents effect behavioural and molecular outcomes	Adult Rat	Yes	Modified weight drop	42
2016	(Xiupeng Xu, Jing Ji)	Sex-related differences in striatal dopaminergic system after traumatic brain injury	Adult CD-1 Mouse	Yes	Trad CCI	43
2017	(William Armstead, Monica Vavilala)	Sex and age differences in epinephrine mechanisms and outcomes after brain injury	Piglet	Yes	FPI	76
2017	(Peter Wirth, Melissa Glenn)	New method to induce mild traumatic brain injury in rodents produces differential outcomes in female and male Sprague Dawley rats	Adult SD Rat	Yes	Impact acceleration novel model	41
2017	(Kristin Free, Anthony Kline)	Comparable impediment of cognitive function in female and male rats subsequent to daily administration of haloperidol after traumatic brain injury	Adult SD Rat	Yes	Trad CCI	31
2017	(Sonia Villapol, Mark Burns)	Sexual dimorphism in the inflammatory response to traumatic brain injury	Adult C57	Yes	Trad CCI	52
2017	(David Wright, Richelle Mychasiuk)	Sex matters: repetitive mild traumatic brain injury in adolescent rats	Adolescent SD	Yes	Modified weight drop	37
2017	(Victor Curvello, William Armstead)	Sex and age differences in phenylephrine mechanisms and outcomes after piglet brain injury	Piglet	Yes	FPI	72
2018	(Amandine Jullienne, Andre Obenaus)	Male and female mice exhibit divergent responses of the cortical vasculature to traumatic brain injury	Adult C57	Yes	Trad CCI	44
2018	(Anna Taylor, Richard Sutton)	Sex differences in thermal, stress, and inflammatory responses to minocycline administration in rats with traumatic brain injury	Adult SD Rat	Yes**	Trad CCI	63
2018	(David Wright, Richelle Mychasiuk)	Telomere length and advanced diffusion MRI as biomarkers for repetitive mild traumatic brain injury in adolescent rats	Adult SD Rat	Yes	Modified weight drop	36

Abbreviations: FPI, fluid percussion injury; SD, Sprague Dawley; Trad CCI, traditional controlled cortical impact; TBI, traumatic brain injury.

*Used animals of same weight, females 5-6 weeks older than Males.

**ovarectomized females.

***pseudopregnant females.

#penetrating injury, very severe and not diffuse.

##compared with males from another study from the same lab.

## Sex-Dependent Changes to Cognition and Behavior Following TBI

In studying the effects of TBI, 3 areas are most commonly assessed for behavioral changes: sensorimotor function, cognitive function, and anxiety/depression-like behavior. Most studies have reported that TBI is associated with reductions in motor performance across multiple tests. Studies that have examined sex have largely reported females to be more resilient than their male counterparts. O’Connor et al investigated the effects of different anesthetics on motor performance after TBI in male and female adult Sprague Dawley (SD) rats. They reported baseline sex differences in rotarod performance, with females outperforming males.^29^ This finding was extended to post-injury performance. Both sexes were impaired following TBI in comparison with pre-injury, but females performed better than males, and their performance recovered at a faster rate. Other studies have also shown that females perform better than males on rotarod and other sensorimotor evaluations such as beam balance and wire grid foot fault.^30–33^ However, in a model of juvenile TBI, no sex differences were reported for sensorimotor tasks.^34–36^ In one of the few studies that reported on a repetitive mTBI model, adolescent female rats were more impaired than males in the beam balance task.^37^ However, a subsequent study from the same group showed no sex difference in the beam balance using the same repetitive model.^36,37^ It is possible that these prepubescent animals showed no differences, or even the opposite association, if sex hormones play a role in producing the behavioral effect. Another study of adolescent C57/Bl6 mice showed progesterone improved performance of males on a wire grip test, but led to worse performance in females, suggesting a ceiling effect.^38^

O’Connor probed spatial memory using the Barnes Maze, and females performed better post injury than males when isofluorane was used as the anesthetic during injury, but no difference between the sexes was detected when halothane was used. This suggests a sex-dependent effect of injury as well as anesthetic. In a subsequent study by the same group, ovariectomized (OVX) SD rats performed similarly to males on rotarod and Barnes maze after injury, but regained their pre-TBI performance advantage over males when treated with progesterone 30 minutes after injury.^39^ Females also were shown to be less impaired in the novel context mismatch task, a test of working memory, after repetitive mTBI.^37^ Other groups have also shown a similar sex-dependent difference in spatial memory using the Morris Water Maze (MWM), where females performed better than males following a controlled cortical impact (CCI).^33^ However, in a previous study from the same group, MWM performance of males and females was similar, but only males improved their performance when housed in an enriched environment.^40^ Still, others have shown the exact opposite association of sex with MWM performance after TBI, with males showing shorter latencies to find the platform.^41^ Of note, this last study was the only one reviewed to use a model that accelerated the animal into a fixed object to produce TBI, rather than an object accelerated into the animal’s head. In summary, cognitive deficits have largely been shown to be more pronounced in male animals than females, but vary across TBI models.

A few groups have examined sex-dependent effects of TBI on depression- and anxiety-like phenotypes using the open field test and the elevated plus maze (EPM). Mychasiuk et al found that both male and female rats exhibit reduced locomotion in the open field following TBI produced using the modified weight drop model, but male locomotion was depressed more than that of females.^42^ They also showed females to have more exploration of the open arms of the EPM than males, indicating greater anxiety in the males, or loss of inhibition in the females. In a subsequent study by the same group, adolescent rats subjected to repetitive mTBI showed sex differences in the forced swim task, where females showed a greater depressive phenotype than males.^37^ Other groups have also found greater activity of female mice in the open field following CCI,^43^ whereas others have found no difference between males and females.^44,45^ The few studies that have reported sex dependence of depression- and anxiety-like behaviors following TBI seem to show a protective effect of female sex, but may this may depend on the TBI model, including number of impacts. The paucity and diversity of studies to date precludes any definitive conclusions.

Finally, one group reported on social behavior in juvenile rats following TBI by using a social play fighting test. Following induction of TBI by modified weight drop, females showed reduced levels of play compared with males, and there were sex-dependent changes in the social interaction of sham animals with TBI animals. Sham females were less likely to interact with TBI females than sham males with their male TBI counterparts.^35^ In a follow-up study, the same group investigated attention deficit hyperactivity disorder–like behavior, following experimental TBI, and found males exhibited reduced inhibitory control compared with females.^45^

## Sex-Dependent Changes to Pathophysiology

Quantifying the extent of tissue damage is important for assessment of TBI pathology. Depending on the severity of TBI, however, frank tissue loss, necrosis, or apoptosis may be absent. In studies of more severe injuries that produce macroscopic brain lesions due to TBI, females consistently exhibit a smaller lesion than males receiving the same TBI, across various species and TBI model mechanisms.^30,31,46–52^ Moreover, in studies of the most severe TBI, females generally have lower mortality than males,^53,54^ with one exception.^55^ Limited reports on neuropathological aspects have detected no divergence of neurodegeneration and cell death by sex, using a silver stain,^56^ while another identified greater apoptosis in males compared with females using TUNEL staining.^57^ Structural magnetic resonance imaging (MRI) studies have also been limited and their results were mixed, such as reduced prefrontal cortex (PFC) volume after mTBI in female SD compared with males in one study and reduced curvature of the corpus callosum in males after repetitive experimental mTBI in another.^36,37^ Lesion recovery after injury was found to diverge between the sexes, demonstrated by labeling with BrdU (bromodeoxyuridine), a marker of newly proliferated neurons. Female mice showed greater cortical, but not hippocampal neurogenesis following CCI than males, indicating regional specificity of the sex dependence.^32^ Further investigation of pathways governing the sex dependence of cell death after TBI has found that X-linked inhibitor of apoptosis (XIAP) is upregulated in female rats after fluid percussion injury (FPI), possibly protecting females from cell death.^58^ Another group showed that male and OVX female rats treated with progesterone after TBI showed reduced expression of caspase-3, a key component of the apoptotic pathway, and produced a reduction in dark cell change in the hippocampus, suggesting less cell death.^39^ Overall, studies indicate females have a protective advantage over males when considering numerous pathologic features such as lesion size, cell death, and recovery/neurogenesis. However, others have shown no sex difference, or an advantage for males over females, effects that appear to be model dependent.^41,44,55^

Deeper investigations into the root causes of sex-dependent injury and recovery of tissue have revealed secondary physiologic changes that may prove informative. Some of the earliest studies of sex dependence investigated TBI-induced edema by measuring cerebral water content. In a series of studies, Roof et al^59,60^ showed that adult SD rats elaborate edema after CCI, with males showing a greater increase than females. This difference was attributed to the action of progesterone, because males who received progesterone 1 hour following injury exhibited levels of edema similar to females.^60^ Another group followed the evolution of post-injury edema attributed to changes in blood brain barrier (BBB) permeability. Male rats elaborated greater edema than females following a weight drop TBI.^61^ In addition, the peak of edema occurred at 5 hours post injury for males, but was delayed to 24 hours for females. Edema resolved by 5 days post injury in females, whereas it persisted in males at 5 days post injury, the last day assayed. Studies from the same group showed administration of estrogen or progesterone 30 minutes after injury reduced brain water content following TBI.^62^ Sex divergence of post-TBI hyperthermia has also been reported. Males remained hyperthermic for 5 days post TBI, whereas females recovered normal body temperatures after 1 day.^63^ Post-injury brain edema and hyperthermia are important prognosticators of impairment^64,65^ that affect males to a greater extent than females.

In association with brain swelling and edema, hypoperfusion with reduction of cerebral blood flow (CBF) is a hallmark of severe TBI that confers significantly worse outcome.^66^ One group has reported sex differences in CBF using the FPI model of TBI in piglets. They were able to show that pial arteries in the parietal cortex constricted in a sex-dependent manner following TBI, where males were more constricted than females.^67^ In subsequent studies, a sex-dependent increase in adrenomedullin was shown to dilate the pial artery to a greater extent in females, by acting on arterial potassium channels.^67,68^ Various pressors were then administered to assess restoration of CBF. Sodium nitroprusside was shown to increase CBF compared with vehicle-treated TBI animals of both sexes but failed to improve outcome.^69^ Phenylephrine was shown to upregulate endothelin-1 (ET-1) in a sex-dependent manner and to act on potassium channels and regulate vasodilation.^70–72^ ET-1 itself was upregulated in a sex-dependent manner following TBI, and phenylephrine further worsened CBF in males, but improved in females.^71,73^ Dopamine was investigated in the same model and blocked TBI-induced upregulation of ET-1 in both males and females, protecting them from hypoperfusion.^74^ Norepinephrine and epinephrine were shown to have differential effects, with norepinephrine augmenting recovery of CBF in females but not males, whereas epinephrine improved CBF for both infants, but failed to confer the same protection for juvenile males.^75,76^ The choice of pressors used to manage cerebral perfusion in severe TBI may be greatly influenced by sex, a finding with important translational implications.

Many groups have reported on the neuroinflammatory response of the brain after TBI, which is recognized as a crucial component of injury, repair, and recovery. However, relatively few studies have reported sex divergence of TBI-related neuroinflammation.^77^ Widespread elevation of the neuroinflammatory markers IL-6, TNFα, and MCP-1 and activated microglia were reported in both sexes following CCI.^78^ When OVX female mice were resupplemented with exogenous estrogen, reduction of proinflammatory IL-6 and MCP-1, an elevation in anti-inflammatory IL-4 was shown in comparison with males, but it made no difference in TBI lesion size.^78^ Taylor et al^63^ also profiled TNFα, IL-6, and IL-1β after TBI, and found female rats expressed more cortical IL-6 than males, but males expressed more cortical TNFα and IL-1β. A study by Villapol et al^52^ did more detailed profiling of microglial morphology and activation state showing male mice exhibited more activated microglia 1 day following CCI than females, as well as greater peripheral macrophage infiltration at 1 and 3 days post CCI. In addition, more astrogliosis was seen by glial fibrillary acidic protein (GFAP) immunofluorescence in males compared with females at 1 and 7 days post CCI in the same study. Jullienne et al,^44^ however, demonstrated greater GFAP expression in female mice at 1 day post CCI but no difference between the sexes at 7 days post injury.

Differences seen in neuroimmune cell populations following injury (above) may have resulted from, and contributed to, differential cytokine expression following injury. Females expressed more IL-1β at 4 hours post injury than males, whereas males expressed more anti-inflammatory TG-Fβ than females at 1 day post injury.^52^ In addition to cytokines, proinflammatory enzymes COX-2 and iNOS play important roles in neuroinflammation and oxidative stress in the brain. Gunther et al^57^ found that TBI induces a greater increase of COX-2 in male rats, and a greater increase in iNOS in females. Although females produced more iNOS, they did not report a sex-dependent difference in oxidative stress by measuring 3-nitrotyrosine. However, Lazarus et al^79^ measured protein carbonylation, a quantifiable consequence of oxidative stress, after CCI and found region-specific increases in males that were significantly greater than females. Although the neuroinflammatory response following experimental TBI is highly dependent on injury model, sex appears to play a crucial role in nature and evolution of the post-TBI neuroinflammatory response.

Few studies have explored sex differences in TBI pathogenesis related to individual genes and pathways associated with brain injury and recovery. Brain-derived neurotrophic factor (BDNF) is an important trophic factor that helps maintain existing neuronal function, and supports new neuron growth, among many other functions.^80^ Chen et al^81^ investigated BDNF expression in adult rats following CCI and found spatial and temporal sex-based differences in BDNF protein levels. Males and females showed significantly different increases in BDNF within frontal cortex ipsilateral to the impact site and in the contralateral hippocampus, respectively, while both males and females showed reductions in BDNF in the ipsilateral hippocampus.

Wagner et al^82^ examined the sex-dependent changes to the dopamine system following TBI and showed the dopamine transporter (DAT) expression to be reduced in cortical-striatal circuits in rats of both sexes, but relatively more so in males.

Hormonal status can have profound effects on behavior and pathology.^83^ To test the effects of TBI on hormone levels in the brain, Meffre et al^84^ performed CCI to produce diffuse TBI in adult rats and measured endogenous hormones by gas chromatography/mass spectrometry. They found increases in progesterone and its metabolites following TBI that varied by sex, location, and time following CCI. The authors suggest that progesterone is reduced into its metabolites locally within the brain following injury, and that it may contribute to differences in recovery between males and females.

## Conclusion

It is still largely unknown what features underlie variation of risk and susceptibility to long-term adverse outcome among concussion patients as well as those who experience repetitive head impacts (RHI). However, epidemiologic and animal studies have given us clues regarding specific pathologies and molecular pathways to explore. The heterogeneous nature of injury complicates the situation, but there remains optimism toward finding better treatment strategies.^85^

At the moment, there is clinical and preclinical evidence that sex may play a role in susceptibility to worse outcomes following brain trauma. Although it is not yet known what aspect of biological sex confers this increased risk, the knowledge of its existence provides a path for investigation into its molecular underpinnings. However, most animal studies have studied males only.^86,87^ In the relatively few studies that have investigated sex in relation to experimental TBI, sex-specific hormones have mainly been the focus. Progesterone has been most commonly associated with beneficial effects, but translation to human studies has not been shown beneficial in any clinical trial.^85^ With the National Institutes of Health (NIH) acknowledging and mandating research address sex as a biological variable, hopefully, the discovery of new mechanisms to target will be expedited.

Although post-concussion syndrome (PCS) can be assessed quite readily in the clinic, it is still very much unclear why some people recover rapidly, but others do not. One aspect that has been consistently shown to exacerbate outcome is repeated injury.^19,88–90^ However, only 2 studies that have examined sex differences in experimental TBI employed more than a single impact.^36,37^ Moreover, a recent systematic review of experimental RHI found all published studies exclusively used male animals.^91^ Studies of human subjects show that PCS is more likely to persist after multiple concussions, and mounting evidence suggests RHI may contribute to long-term disease and dysfunction, even in the absence of recognized concussion.^92–94^ In light of these clinical realities, there seems to be some degree of disconnect from current preclinical research approaches.^3,92,95^ Uncovering mechanisms underlying RHI pathology and dysfunction is an imperative goal because of the tremendous “window of opportunity” that exists to treat the disease, or possibly prevent it altogether. Identifying risk factors on the population and molecular level should provide legitimate targets to intervene and steer at-risk individuals toward recovery from head trauma.

To progress toward these goals, we suggest the following:

- Inclusion of both sexes in all preclinical TBI research to determine sex dependence of injury and recovery.
- Increased use of newer and more translatable models for mild TBI that do not require craniotomy and direct brain impact.
- Increased focus on multiple impact models to reveal how behavioral and pathophysiological sequelae differ from isolated concussion.
- Increased use of advanced imaging techniques to facilitate reverse translation of the human condition into animal models for more robust and relevant findings.

## References

[1] Ma VY Chan L Carruthers KJ. Incidence, prevalence, costs, and impact on disability of common conditions requiring rehabilitation in the United States: stroke, spinal cord injury, traumatic brain injury, multiple sclerosis, osteoarthritis, rheumatoid arthritis, limb loss, and back pain. Arch Phys Med Rehabil. 2014;95:986–995.e1. 24462839 10.1016/j.apmr.2013.10.032 PMC4180670

[2] Langlois JA Rutland-Brown W Wald MM. The epidemiology and impact of traumatic brain injury: a brief overview. J Head Trauma Rehabil. 2006;21:375–378. 16983222 10.1097/00001199-200609000-00001

[3] Gardner RC Yaffe K. Epidemiology of mild traumatic brain injury and neurodegenerative disease. Mol Cell Neurosci. 2015;66:75–80. 25748121 10.1016/j.mcn.2015.03.001 PMC4461453

[4] Taylor CA Bell JM Breiding MJ Xu L. Traumatic brain injury-related emergency department visits, hospitalizations, and deaths—United States, 2007 and 2013. MMWR Surveill Summ. 2017;66:1–16. 10.15585/mmwr.ss6609a1 PMC5829835 28301451

[5] Centers for Disease Control Prevention (CDC) National Center for Injury Prevention and Control. Report to congress on mild traumatic brain injury in the United States: steps to prevent a serious public health problem. Atlanta, GA: Centers for Disease Control and Prevention; 2003.

[6] Gardner AJ Zafonte R. Neuroepidemiology of traumatic brain injury. Handb Clin Neurol. 2016;138:207–223. 27637960 10.1016/B978-0-12-802973-2.00012-4

[7] Kushner D. Mild traumatic brain injury: toward understanding manifestations and treatment. Arch Intern Med. 1998;158:1617–1624. 9701095 10.1001/archinte.158.15.1617

[8] Halstead ME Walter KD Moffatt K; Council on Sports Medicine and Fitness. Clinical report—sport-related concussion in children and adolescents. Pediatrics. 2010;126:597–615. 20805152 10.1542/peds.2010-2005

[9] Shenton ME Hamoda HM Schneiderman JS et al A review of magnetic resonance imaging and diffusion tensor imaging findings in mild traumatic brain injury. Brain Imaging Behav. 2012;6:137–192. 22438191 10.1007/s11682-012-9156-5 PMC3803157

[10] Rosenbaum SB Lipton ML. Embracing chaos: the scope and importance of clinical and pathological heterogeneity in mTBI. Brain Imaging Behav. 2012;6:255–282. 22549452 10.1007/s11682-012-9162-7

[11] McCrea M Guskiewicz KM Marshall SW et al Acute effects and recovery time following concussion in collegiate football players: the NCAA concussion study. JAMA. 2003;290:2556–2563. 14625332 10.1001/jama.290.19.2556

[12] Karr JE Areshenkoff CN Garcia-Barrera MA. The neuropsychological outcomes of concussion: a systematic review of meta-analyses on the cognitive sequelae of mild traumatic brain injury. Neuropsychology. 2014;28:321–336. 24219611 10.1037/neu0000037

[13] Alexander MP. Mild traumatic brain injury: pathophysiology, natural history, and clinical management. Neurology. 1995;45:1253–1260. 7617178 10.1212/wnl.45.7.1253

[14] Helmy A De Simoni MG Guilfoyle MR Carpenter KL Hutchinson PJ. Cytokines and innate inflammation in the pathogenesis of human traumatic brain injury. Prog Neurobiol. 2011;95:352–372. 21939729 10.1016/j.pneurobio.2011.09.003

[15] Mouzon BC Bachmeier C Ferro A et al Chronic neuropathological and neurobehavioral changes in a repetitive mild traumatic brain injury model. Ann Neurol. 2014;75:241–254. 24243523 10.1002/ana.24064

[16] Tsitsopoulos PP Marklund N. Amyloid-beta peptides and tau protein as biomarkers in cerebrospinal and interstitial fluid following traumatic brain injury: a review of experimental and clinical studies. Front Neurol. 2013;4:79. 23805125 10.3389/fneur.2013.00079 PMC3693096

[17] Thomas DG Apps JN Hoffmann RG McCrea M Hammeke T. Benefits of strict rest after acute concussion: a randomized controlled trial. Pediatrics. 2015;135:213–223. 25560444 10.1542/peds.2014-0966

[18] McCrory P Meeuwisse W Dvorak J et al Consensus statement on concussion in sport—the 5(th) international conference on concussion in sport held in Berlin, October 2016. Br J Sports Med. 2017;51:838–847. 28446457 10.1136/bjsports-2017-097699

[19] Asken BM Hack DC McCrea MA. The modern landscape of sport-related concussion research: key achievements and future directions. Handb Clin Neurol. 2018;158:269–278. 30482355 10.1016/B978-0-444-63954-7.00026-4

[20] Preiss-Farzanegan SJ Chapman B Wong TM Wu J Bazarian JJ. The relationship between gender and postconcussion symptoms after sport-related mild traumatic brain injury. PM R. 2009;1:245–253. 19627902 10.1016/j.pmrj.2009.01.011 PMC5237580

[21] Broshek DK Kaushik T Freeman JR Erlanger D Webbe F Barth JT. Sex differences in outcome following sports-related concussion. J Neurosurg. 2005;102:856–863. 15926710 10.3171/jns.2005.102.5.0856

[22] Bazarian JJ Blyth B Mookerjee S He H McDermott MP. Sex differences in outcome after mild traumatic brain injury. J Neurotrauma. 2010;27:527–539. 19938945 10.1089/neu.2009.1068 PMC2867588

[23] Berz K Divine J Foss KB Heyl R Ford KR Myer GD. Sex-specific differences in the severity of symptoms and recovery rate following sports-related concussion in young athletes. Phys Sportsmed. 2013;41:58–63. 10.3810/psm.2013.05.2015 23703518

[24] Carroll LJ Cassidy JD Peloso PM et al Prognosis for mild traumatic brain injury: results of the WHO collaborating centre task force on mild traumatic brain injury. J Rehabil Med. 2004:84–105. 10.1080/16501960410023859 15083873

[25] Cassidy JD Carroll LJ Peloso PM et al Incidence, risk factors and prevention of mild traumatic brain injury: results of the WHO collaborating centre task force on mild traumatic brain injury. J Rehabil Med. 2004;43:28–60. 10.1080/16501960410023732 15083870

[26] Laker SR. Epidemiology of concussion and mild traumatic brain injury. PM R. 2011;3:S354–S358. 22035677 10.1016/j.pmrj.2011.07.017

[27] Caccese JB Buckley TA Tierney RT et al Head and neck size and neck strength predict linear and rotational acceleration during purposeful soccer heading. Sports Biomech. 2017;17:1–15. 29037111 10.1080/14763141.2017.1360385

[28] Catenaccio E Mu W Kaplan A et al Characterization of neck strength in healthy young adults. PM R. 2017;9:884–891. 28167302 10.1016/j.pmrj.2017.01.005 PMC5545075

[29] O’Connor CA Cernak I Vink R. Interaction between anesthesia, gender, and functional outcome task following diffuse traumatic brain injury in rats. J Neurotrauma. 2003;20:533–541. 12906738 10.1089/089771503767168465

[30] Geddes RI Peterson BL Stein DG Sayeed I. Progesterone treatment shows benefit in female rats in a pediatric model of controlled cortical impact injury. PLoS ONE. 2016;11:e0146419. 26799561 10.1371/journal.pone.0146419 PMC4723082

[31] Free KE Greene AM Bondi CO Lajud N de la Tremblaye PB Kline AE. Comparable impediment of cognitive function in female and male rats subsequent to daily administration of haloperidol after traumatic brain injury. Exp Neurol. 2017;296:62–68. 28698031 10.1016/j.expneurol.2017.07.004 PMC5557279

[32] Xiong Y Mahmood A Lu D et al Role of gender in outcome after traumatic brain injury and therapeutic effect of erythropoietin in mice. Brain Res. 2007;1185:301–312. 17976541 10.1016/j.brainres.2007.09.052 PMC2219338

[33] Wagner AK Kline AE Ren D et al Gender associations with chronic methylphenidate treatment and behavioral performance following experimental traumatic brain injury. Behav Brain Res. 2007;181:200–209. 17517440 10.1016/j.bbr.2007.04.006 PMC1974874

[34] Russell KL Kutchko KM Fowler SC Berman NE Levant B. Sensorimotor behavioral tests for use in a juvenile rat model of traumatic brain injury: assessment of sex differences. J Neurosci Methods. 2011;199:214–222. 21600923 10.1016/j.jneumeth.2011.05.008 PMC3142868

[35] Mychasiuk R Hehar H Farran A Esser MJ. Mean girls: sex differences in the effects of mild traumatic brain injury on the social dynamics of juvenile rat play behaviour. Behav Brain Res. 2014;259:284–291. 24231261 10.1016/j.bbr.2013.10.048

[36] Wright DK O’Brien TJ Mychasiuk R Shultz SR. Telomere length and advanced diffusion MRI as biomarkers for repetitive mild traumatic brain injury in adolescent rats. Neuroimage Clin. 2018;18:315–324. 29876252 10.1016/j.nicl.2018.01.033 PMC5987845

[37] Wright DK O’Brien TJ Shultz SR Mychasiuk R. Sex matters: repetitive mild traumatic brain injury in adolescent rats. Ann Clin Transl Neurol. 2017;4:640–654. 28904986 10.1002/acn3.441 PMC5590540

[38] Mannix R Berglass J Berkner J et al Sex differences in the effect of progesterone after controlled cortical impact in adolescent mice: a preliminary study. J Neurosurg. 2014;121:1337–1341. 25280093 10.3171/2014.8.JNS14715 PMC5632951

[39] O’Connor CA Cernak I Johnson F Vink R. Effects of progesterone on neurologic and morphologic outcome following diffuse traumatic brain injury in rats. Exp Neurol. 2007;205:145–153. 17362936 10.1016/j.expneurol.2007.01.034

[40] Wagner AK Kline AE Sokoloski J Zafonte RD Capulong E Dixon CE. Intervention with environmental enrichment after experimental brain trauma enhances cognitive recovery in male but not female rats. Neurosci Lett. 2002;334:165–168. 12453621 10.1016/s0304-3940(02)01103-5

[41] Wirth P Yu W Kimball AL Liao J Berkner P Glenn MJ. New method to induce mild traumatic brain injury in rodents produces differential outcomes in female and male Sprague Dawley rats. J Neurosci Methods. 2017;290:133–144. 28780369 10.1016/j.jneumeth.2017.07.030 PMC5790318

[42] Mychasiuk R Hehar H Candy S Ma I Esser MJ. The direction of the acceleration and rotational forces associated with mild traumatic brain injury in rodents effect behavioural and molecular outcomes. J Neurosci Methods. 2016;257:168–178. 26484783 10.1016/j.jneumeth.2015.10.002

[43] Xu X Cao S Chao H Liu Y Ji J. Sex-related differences in striatal dopaminergic system after traumatic brain injury. Brain Res Bull. 2016;124:214–221. 27210290 10.1016/j.brainresbull.2016.05.010

[44] Jullienne A Salehi A Affeldt B et al Male and female mice exhibit divergent responses of the cortical vasculature to traumatic brain injury. J Neurotrauma. 2018;35:1646–1658. 29648973 10.1089/neu.2017.5547 PMC6016102

[45] Mychasiuk R Hehar H Esser MJ. A mild traumatic brain injury (mTBI) induces secondary attention-deficit hyperactivity disorder-like symptomology in young rats. Behav Brain Res. 2015;286:285–292. 25771208 10.1016/j.bbr.2015.03.010

[46] Igarashi T Huang TT Noble LJ. Regional vulnerability after traumatic brain injury: gender differences in mice that overexpress human copper, zinc superoxide dismutase. Exp Neurol. 2001;172:332–341. 11716557 10.1006/exnr.2001.7820

[47] Suzuki T Bramlett HM Dietrich WD. The importance of gender on the beneficial effects of posttraumatic hypothermia. Exp Neurol. 2003;184:1017–1026. 14769396 10.1016/S0014-4886(03)00389-3

[48] Jones NC Constantin D Prior MJ Morris PG Marsden CA Murphy S. The neuroprotective effect of progesterone after traumatic brain injury in male mice is independent of both the inflammatory response and growth factor expression. Eur J Neurosci. 2005;21:1547–1554. 15845082 10.1111/j.1460-9568.2005.03995.x

[49] Qu C Lu D Goussev A Schallert T Mahmood A Chopp M. Effect of atorvastatin on spatial memory, neuronal survival, and vascular density in female rats after traumatic brain injury. J Neurosurg. 2005;103:695–701. 16266052 10.3171/jns.2005.103.4.0695

[50] Missios S Harris BT Dodge CP et al Scaled cortical impact in immature swine: effect of age and gender on lesion volume. J Neurotrauma. 2009;26:1943–1951. 19469691 10.1089/neu.2009.0956 PMC2822800

[51] Robertson CL Saraswati M. Progesterone protects mitochondrial function in a rat model of pediatric traumatic brain injury. J Bioenerg Biomembr. 2015;47:43–51. 25348484 10.1007/s10863-014-9585-5

[52] Villapol S Loane DJ Burns MP. Sexual dimorphism in the inflammatory response to traumatic brain injury. Glia. 2017;65:1423–1438. 28608978 10.1002/glia.23171 PMC5609840

[53] Roof RL Hall ED Estrogen-related gender difference in survival rate and cortical blood flow after impact-acceleration head injury in rats. J Neurotrauma. 2000;17:1155–1169. 11186229 10.1089/neu.2000.17.1155

[54] Kupina NC Detloff MR Bobrowski WF Snyder BJ Hall ED Cytoskeletal protein degradation and neurodegeneration evolves differently in males and females following experimental head injury. Exp Neurol. 2003;180:55–73. 12668149 10.1016/s0014-4886(02)00048-1

[55] Emerson CS Headrick JP Vink R. Estrogen improves biochemical and neurologic outcome following traumatic brain injury in male rats, but not in females. Brain Research. 1993;608:95–100. 8495351 10.1016/0006-8993(93)90778-l

[56] Hall ED Gibson TR Pavel KM. Lack of a gender difference in post-traumatic neurodegeneration in the mouse controlled cortical impact injury model. J Neurotrauma. 2005;22:669–679. 15941376 10.1089/neu.2005.22.669

[57] Gunther M Plantman S Davidsson J Angeria M Mathiesen T Risling M. COX-2 regulation and TUNEL-positive cell death differ between genders in the secondary inflammatory response following experimental penetrating focal brain injury in rats. Acta Neurochir (Wien). 2015;157:649–659. 25597483 10.1007/s00701-014-2331-2

[58] Bramlett HM Furones-Alonso O Lotocki G Rodriguez-Paez A Sanchez-Molano J Keane RW. Sex differences in XIAP cleavage after traumatic brain injury in the rat. Neurosci Lett. 2009;461:49–53. 19500649 10.1016/j.neulet.2009.05.071 PMC2716731

[59] Roof RL Duvdevani R Stein DG. Gender influences outcome of brain injury: progesterone plays a protective role. Brain Res. 1993;607:333–336. 8481809 10.1016/0006-8993(93)91526-x

[60] Roof RL Duvdevani R Heyburn JW Stein DG. Progesterone rapidly decreases brain edema: treatment delayed up to 24 hours is still effective. Exp Neurol. 1996;138:246–251. 8620923 10.1006/exnr.1996.0063

[61] O’Connor CA Cernak I Vink R. The temporal profile of edema formation differs between male and female rats following diffuse traumatic brain injury. Acta Neurochir Suppl. 2006;96:121–124. 16671438 10.1007/3-211-30714-1_27

[62] O’Connor CA Cernak I Vink R. Both estrogen and progesterone attenuate edema formation following diffuse traumatic brain injury in rats. Brain Res. 2005;1062:171–174. 16256079 10.1016/j.brainres.2005.09.011

[63] Taylor AN Tio DL Paydar A Sutton RL. Sex differences in thermal, stress, and inflammatory responses to minocycline administration in rats with traumatic brain injury. J Neurotrauma. 2018;35:630–638. 29179648 10.1089/neu.2017.5238

[64] Feickert HJ Drommer S Heyer R. Severe head injury in children: impact of risk factors on outcome. J Trauma. 1999;47:33–38. 10421183 10.1097/00005373-199907000-00008

[65] Marmarou A. Traumatic brain edema: an overview. Acta Neurochir Suppl (Wien). 1994;60:421–424. 7976607 10.1007/978-3-7091-9334-1_114

[66] Coates BM Vavilala MS Mack CD et al Influence of definition and location of hypotension on outcome following severe pediatric traumatic brain injury. Crit Care Med. 2005;33:2645–2650. 16276192 10.1097/01.ccm.0000186417.19199.9b PMC1361352

[67] Armstead WM Vavilala MS. Adrenomedullin reduces gender-dependent loss of hypotensive cerebrovasodilation after newborn brain injury through activation of ATP-dependent K channels. J Cereb Blood Flow Metab. 2007;27:1702–1709. 17377515 10.1038/sj.jcbfm.9600473

[68] Armstead WM Kiessling JW Bdeir K Kofke WA Vavilala MS. Adrenomedullin prevents sex-dependent impairment of autoregulation during hypotension after piglet brain injury through inhibition of ERK MAPK upregulation. J Neurotrauma. 2010;27:391–402. 20170313 10.1089/neu.2009.1094 PMC2834454

[69] Armstead WM Kiessling JW Kofke WA Vavilala MS. SNP improves cerebral hemodynamics during normotension but fails to prevent sex dependent impaired cerebral autoregulation during hypotension after brain injury. Brain Res. 2010;1330:142–150. 20298682 10.1016/j.brainres.2010.03.024 PMC2860054

[70] Armstead WM Kiessling JW Riley J Kofke WA Vavilala MS. Phenylephrine infusion prevents impairment of ATP- and calcium-sensitive potassium channel-mediated cerebrovasodilation after brain injury in female, but aggravates impairment in male, piglets through modulation of ERK MAPK upregulation. J Neurotrauma. 2011;28:105–111. 20964536 10.1089/neu.2010.1581 PMC3019588

[71] Armstead WM Riley J Vavilala MS. TBI sex dependently upregulates ET-1 to impair autoregulation, which is aggravated by phenylephrine in males but is abrogated in females. J Neurotrauma. 2012;29:1483–1490. 22335188 10.1089/neu.2011.2248 PMC3335106

[72] Curvello V Hekierski H Riley J Vavilala M Armstead WM. Sex and age differences in phenylephrine mechanisms and outcomes after piglet brain injury. Pediatr Res. 2017;82:108–113. 28355201 10.1038/pr.2017.83 PMC5509507

[73] Armstead WM Kiessling JW Kofke WA Vavilala MS. Impaired cerebral blood flow autoregulation during posttraumatic arterial hypotension after fluid percussion brain injury is prevented by phenylephrine in female but exacerbated in male piglets by extracellular signal-related kinase mitogen-activated protein kinase upregulation. Crit Care Med. 2010;38:1868–1874. 20562700 10.1097/CCM.0b013e3181e8ac1a PMC3541517

[74] Armstead WM Riley J Vavilala MS. Dopamine prevents impairment of autoregulation after traumatic brain injury in the newborn pig through inhibition of up-regulation of endothelin-1 and extracellular signal-regulated kinase mitogen-activated protein kinase. Pediatr Crit Care Med. 2013;14:11. 10.1097/PCC.0b013e3182712b44 PMC3567252 23314184

[75] Armstead WM Riley J Vavilala MS. Preferential protection of cerebral autoregulation and reduction of hippocampal necrosis with norepinephrine after traumatic brain injury in female piglets. Pediatr Crit Care Med. 2016;17:e130–e137. 26741414 10.1097/PCC.0000000000000603 PMC4779739

[76] Armstead WM Riley J Vavilala MS. Sex and age differences in epinephrine mechanisms and outcomes after brain injury. J Neurotrauma. 2017;34:1666–1675. 27912253 10.1089/neu.2016.4770 PMC5397223

[77] Kumar A Loane DJ. Neuroinflammation after traumatic brain injury: opportunities for therapeutic intervention. Brain Behav Immun. 2012;26:1191–1201. 22728326 10.1016/j.bbi.2012.06.008

[78] Bruce-Keller AJ Dimayuga FO Reed JL et al Gender and estrogen manipulation do not affect traumatic brain injury in mice. J Neurotrauma. 2007;24:203–215. 17263684 10.1089/neu.2006.0163

[79] Lazarus RC Buonora JE Jacobowitz DM Mueller GP. Protein carbonylation after traumatic brain injury: cell specificity, regional susceptibility, and gender differences. Free Radic Biol Med. 2015;78:89–100. 25462645 10.1016/j.freeradbiomed.2014.10.507

[80] Gray JD Milner TA McEwen BS. Dynamic plasticity: the role of glucocorticoids, brain-derived neurotrophic factor and other trophic factors. Neuroscience. 2013;239:214–227. 22922121 10.1016/j.neuroscience.2012.08.034 PMC3743657

[81] Chen X Li Y Kline AE Dixon CE Zafonte RD Wagner AK. Gender and environmental effects on regional brain-derived neurotrophic factor expression after experimental traumatic brain injury. Neuroscience. 2005;135:11–17. 16084663 10.1016/j.neuroscience.2005.05.041

[82] Wagner AK Chen X Kline AE Li Y Zafonte RD Dixon CE. Gender and environmental enrichment impact dopamine transporter expression after experimental traumatic brain injury. Exp Neurol. 2005;195:475–483. 16023635 10.1016/j.expneurol.2005.06.009

[83] McEwen BS Milner TA. Understanding the broad influence of sex hormones and sex differences in the brain. J Neurosci Res. 2017;95:24–39. 27870427 10.1002/jnr.23809 PMC5120618

[84] Meffre D Pianos A Liere P et al Steroid profiling in brain and plasma of male and pseudopregnant female rats after traumatic brain injury: analysis by gas chromatography/mass spectrometry. Endocrinology. 2007;148:2505–2517. 17303653 10.1210/en.2006-1678

[85] Stein DG Geddes RI Sribnick EA. Recent developments in clinical trials for the treatment of traumatic brain injury. Handb Clin Neurol. 2015;127:433–451. 25702233 10.1016/B978-0-444-52892-6.00028-3

[86] Babikian T Prins ML Cai Y et al Molecular and physiological responses to juvenile traumatic brain injury: focus on growth and metabolism. Dev Neurosci. 2010;32:431–441. 21071915 10.1159/000320667 PMC3215243

[87] Prins ML Hales A Reger M Giza CC Hovda DA. Repeat traumatic brain injury in the juvenile rat is associated with increased axonal injury and cognitive impairments. Dev Neurosci. 2010;32:510–518. 20829578 10.1159/000316800 PMC3215244

[88] Broglio SP McCrea M McAllister T et al A national study on the effects of concussion in collegiate athletes and US military service academy members: the NCAA-DoD concussion assessment, research and education (CARE) consortium structure and methods. Sports Med. 2017;47:1437–1451. 28281095 10.1007/s40279-017-0707-1 PMC5488134

[89] Guskiewicz KM McCrea M Marshall SW et al Cumulative effects associated with recurrent concussion in collegiate football players: the NCAA Concussion Study. JAMA. 2003;290:2549–2555. 14625331 10.1001/jama.290.19.2549

[90] Guskiewicz KM Marshall SW Bailes J et al Recurrent concussion and risk of depression in retired professional football players. Med Sci Sports Exerc. 2007;39:903–909. 17545878 10.1249/mss.0b013e3180383da5

[91] Hoogenboom WS Branch CA Lipton ML Animal models of closed-skull, repetitive mild traumatic brain injury [published online ahead of print February 26, 2019]. Pharmacol Ther. doi:10.1016/j.pharmthera.2019.02.016. PMC6536340 30822463

[92] Gavett BE Stern RA McKee AC. Chronic traumatic encephalopathy: a potential late effect of sport-related concussive and subconcussive head trauma. Clin Sports Med. 2011;30:179.xi. 21074091 10.1016/j.csm.2010.09.007 PMC2995699

[93] Huber BR Alosco ML Stein TD McKee AC. Potential long-term consequences of concussive and subconcussive injury. Phys Med Rehabil Clin N Am. 2016;27:503–511. 27154859 10.1016/j.pmr.2015.12.007 PMC4866819

[94] Bahrami N Sharma D Rosenthal S et al Subconcussive head impact exposure and white matter tract changes over a single season of youth football. Radiology. 2016:919–926. 10.1148/radiol.2016160564 PMC5131834 27775478

[95] Bailes JE Petraglia AL Omalu BI Nauman E Talavage T. Role of subconcussion in repetitive mild traumatic brain injury. J Neurosurg. 2013;119:1235–1245. 23971952 10.3171/2013.7.JNS121822

